# Cutaneous Melanoma in Alpine Population: Incidence Trends and Clinicopathological Profile

**DOI:** 10.3390/curroncol29030175

**Published:** 2022-03-21

**Authors:** Alessandra Buja, Massimo Rugge, Giuseppe De Luca, Emanuela Bovo, Manuel Zorzi, Chiara De Toni, Claudia Cozzolino, Antonella Vecchiato, Paolo Del Fiore, Romina Spina, Sandro Cinquetti, Vincenzo Baldo, Carlo Riccardo Rossi, Simone Mocellin

**Affiliations:** 1 Department of Cardiologic, Vascular and Thoracic Sciences and Public Health, University of Padua, 35131 Padua, Italy; giuseppe.deluca.2@studenti.unipd.it (G.D.L.); vincenzo.baldo@unipd.it (V.B.); 2 Pathology and Cytopathology Unit, Department of Medicine-DIMED, University of Padua, 35131 Padua, Italy; massimo.rugge@unipd.it; 3 Azienda Zero, Veneto Tumor Registry (RTV), 35131 Padua, Italy; emanuela.bovo@azero.veneto.it (E.B.); manuel.zorzi@azero.veneto.it (M.Z.); 4 Department of Statistical Sciences, University of Padua, 35131 Padua, Italy; chiara.detoni.1@studenti.unipd.it; 5 Soft-Tissue, Peritoneum and Melanoma Surgical Oncology Unit, Veneto Institute of Oncology IOV-IRCCS, 35131 Padua, Italy; claudia.cozzolino@iov.veneto.it (C.C.); antonella.vecchiato@iov.veneto.it (A.V.); carlor.rossi@unipd.it (C.R.R.); simone.mocellin@iov.veneto.it (S.M.); 6 Department of Surgery, Oncology and Gastroenterology (DISCOG), University of Padua, 35131 Padua, Italy; paolo.delfiore@iov.veneto.it (P.D.F.); romina.spina@iov.veneto.it (R.S.); 7 Hygiene and Public Health Service (SISP), Azienda ULSS 1 Dolomiti, 32100 Belluno, Italy; sandro.cinquetti@aulss1.veneto.it

**Keywords:** melanoma, incidence, joinpoint, mountain residents, ultraviolet radiation, UVR, spatial analysis, temporal trends, sun exposure

## Abstract

Previous studies associated high-level exposure to ultraviolet radiation with a greater risk of cutaneous malignant melanoma (CMM). This study focuses on the changing incidence of CMM over time (from 1990 to 2017) in the Veneto region of Northeast Italy, and its Alpine area (the province of Belluno). The clinicopathological profile of CMM by residence is also considered. A joinpoint regression analysis was performed to identify significant changes in the yearly incidence of CMM by sex and age. For each trend, the average annual percent change (AAPC) was also calculated. In the 2017 CMM cohort, the study includes a descriptive analysis of the disease’s categorical clinicopathological variables. In the population investigated, the incidence of CMM has increased significantly over the last 30 years. The AAPC in the incidence of CMM was significantly higher among Alpine residents aged 0–49 than for the rest of the region’s population (males: 6.9 versus 2.4; females 7.7 versus 2.7, respectively). Among the Alpine residents, the AAPC was 3.35 times greater for females aged 0–49 than for people aged 50+. The clinicopathological profile of CMM was significantly associated with the place of residence. Over three decades, the Veneto population has observed a significant increase in the incidence of CMM, and its AAPC. Both trends have been markedly more pronounced among Alpine residents, particularly younger females. While epidemiology and clinicopathological profiles support the role of UV radiation in CMM, the young age of this CMM-affected female population points to other possible host-related etiological factors. These findings also confirm the importance of primary and secondary prevention strategies.

## 1. Introduction

Between 2006 and 2016, the worldwide incidence of cutaneous malignant melanoma (CMM) increased by about 39%, particularly among Caucasian populations [1,2]. In Italy, the incidence of CMM increased consistently between 1990 and 2015, in both sexes (from 1.6/10 to 21/10 in males, and from 2/10 to 17/10 in females) [3]. Excluding non-melanoma skin cancers, CMM ranked among the 10 most frequently occurring malignancies worldwide in 2020, with an overall 5-year prevalence of 70 per 10 [4,5]. Such a burden of disease has raised other major clinical concerns [5], and has also contributed to rising health-related social costs [6,7,8].

The risk factors for CMM include fair skin and hair, family history (including genetic susceptibility), and exposure to ultraviolet radiation (UVR), particularly from the sun (UV-A and UV-B) [9]. Stratospheric ozone and cloud cover both have a strong impact on long-term ground-level UV radiation. Stratospheric ozone depletion may increase human exposure to UV-B, which results in DNA damage, etiologically involved in both melanomatous and non-melanomatous skin cancers [10,11,12,13]. UV-A radiation can also damage DNA repair systems, thereby promoting cancer cell invasion and anticancer immune response [14]. Recent data regarding the European continent have revealed an increase in the occurrence of mini ozone holes [15,16], particularly affecting extensive areas of the middle European mountain regions [17], where exposure to UVR is estimated to increase by 10–12% per 1000 m of altitude [18].

Previous studies focused on the causative relationships between UVR exposure and the epidemiological and biological profile of CMM [19,20,21,22]. Based on the large body of evidence accumulated, histology can distinguish between two main etiological subgroups of CMM, one “typically” and the other “not consistently” associated with “cumulative solar damage” [23].

This retrospective population-based study addresses the temporal trends in the incidence of CMM (from 1990 to 2017) in the Veneto region of Northeastern Italy, particularly focusing on the Alpine province of Belluno. The study also compares the clinicopathological profile of CMM occurring in the region’s residents living in the mountains as opposed to those living elsewhere.

## 2. Materials and Methods

### 2.1. Context and Data Sources

Veneto is a region in Northeastern Italy, covering about 18,399 square kilometers. It is the fifth largest Italian region, with a population of 4.9 million. One of the region’s provinces, Belluno (population 201,309 [24]), is in the Alps, with an average altitude (a.a.) of 773 m above sea level, whereas the a.a. of the other provinces ranges between 210 (Vicenza) and 6 m (Venice).

Consistently with Italy’s national public healthcare system, the Veneto region delivers healthcare based on the fundamental values of universality, free access, freedom of choice, pluralism in provision, and equity. It focuses strongly on meeting the needs of individuals and the community through the integration of health and social services [25].

This retrospective population-based study draws on epidemiological data regarding CMM collected by the Veneto Regional Tumor Registry for the years 1990–2017. The temporal trends in the incidence of the disease (standardized for the European population in 2013) were calculated by age group and sex. The CMM incidence rates were considered for 2017, both in the region as a whole and in the Alpine province of Belluno. The clinicopathological profiles of the 2017 incident CMM cases were also compared, by patients’ place of residence (mountain versus elsewhere), considering the following variables: age, sex, primary cancer site (face, limbs, lower back, or trunk), pathological tumor stage (pTNM), histological subtype, growth pattern (radial versus vertical), Breslow thickness, ulceration, tumor-infiltrating lymphocytes (TILs; present versus absent), and cancer regression (present versus absent) [26].

### 2.2. Statistics

A joinpoint regression analysis was performed to identify significant changes in the yearly trends of the standardized incidence rates in the European population, stratified by sex and age group [27]. For each of the trends identified, the annual percent change (APC) was also calculated by fitting a regression line to the natural logarithm of the rates, using calendar year as a regression variable. Using the Joinpoint Regression Program 4.3, the average annual percent change (AAPC) was calculated (based on an underlying joinpoint model) as the geometric weighted average of the APCs, with the weights equating to the length of each time interval segment.

A descriptive analysis specifically addressed the 2017 CMM cohort, using absolute and relative frequencies for the categorical variables. The associations between CMM patients’ place of residence and their clinicopathological variables were tested with Pearson’s chi-squared or Fisher tests [28] and Student’s [29] *t*-tests, comparing the populations of the Alpine and Veneto regions, excluding Belluno. RStudio software was used for this analysis [30].

### 2.3. Ethics

The data analysis was performed on anonymized aggregate data, with no chance of individuals being identifiable. The study complied with the Declaration of Helsinki and with resolution n. 9/2016 of the Italian Data Protection Authority. Ethical approval for the study was obtained from the Veneto Oncological Institute’s Ethics Committee (No. 52/2016).

## 3. Results

**Figure 1:**
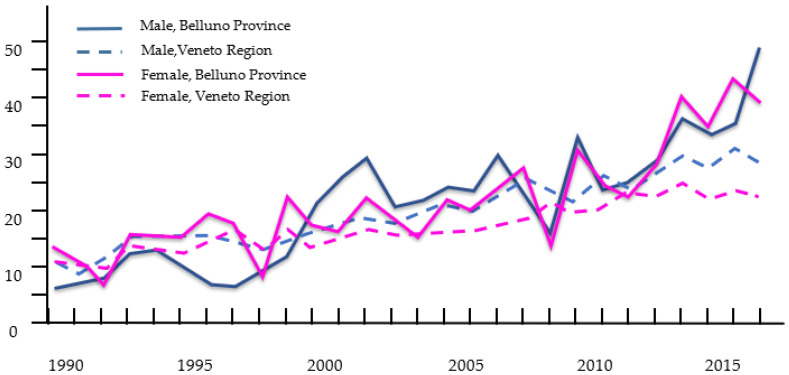
Years 1990–2017. Temporal trend of CMM incidence rate by sex. Comparison between Veneto regional (Veneto) and Alpen Belluno province populations.

Figure 1 shows the trend of the CMM incidence rates by sex from 1990 to 2017, in the whole Veneto population and in the residents of the Alpine province of Belluno. Over the years, there was a significant difference between the APCs in the incidence of CMM in Belluno versus the other Veneto provinces, for both sexes. In the Alpine population, the AAPC was significantly higher for females (females: 4.4 versus 2.8; males: 5.7 versus 3.5, respectively; Table 1).

Considering the AAPC values stratified by sex and age group (Table 1), in the regional population as a whole, the AAPC for males under 50 years old was significantly lower than for older men (2.4 versus 3.9, respectively); among females, there were no significant age-related differences in the AAPC. Alpine residents (of both sexes) under 50 years old had significantly higher AAPCs than in the rest of the regional population, and this difference was greater for females (females: 7.7 versus 2.7; males: 6.9 versus 2.4, respectively). No significant differences emerged between people aged 50 or more living in the mountains, as opposed to elsewhere in the region (females: 2.3 versus 2.8; males: 5.1 versus 3.9, respectively). There was a significant difference in the AAPC of the female residents of Belluno under 50 years old, as opposed to those over 50 years old (7.7 versus 2.3, respectively).

Table 2 shows the clinicopathological profiles of the cases of CMM obtained from the high-resolution regional cancer registry (RTV). The CMMs in patients living in the Alpine province differed, in terms of primary site and histological subtype. In particular, the face was more often involved in the Alpine population (20.21% versus 10.94% elsewhere), while CMM of the trunk was more common in other provinces (Alpine population: 5.3% versus 13.1% elsewhere). Superficial spreading melanoma was the most prevalent histotype in both the region as a whole and the Alpine population, while the proportion of lentigo malignant melanoma was three times higher in the mountains than elsewhere in the region (6.1% versus 2.05%, respectively). Cases of TILs and cancer regression were significantly more common in CMM patients living in the mountains.

## 4. Discussion

This study identified a significantly rising trend over time in the incidence of CMM in both sexes of the population in the Veneto region, and the increase was even more significant among people living in the Alpine province of Belluno. This clear epidemiological trend is consistent with the results of previous retrospective studies conducted in mountain areas, and it is plausibly attributable to a greater exposure to UVR [9,18]. The risk associated with UVR exposure can also be dynamically influenced by the regional atmosphere and weather phenomena, such as ozone mini holes and cloud cover [12]. An Austrian study by Haluza and coworkers produced similar findings, observing an increased melanoma incidence for inhabitants living at higher altitudes [17]. Núñez-González et al. also recently reported a significantly greater increase in the temporal trends of CMM mortality among highland populations in Ecuador, compared with people living on the coast, or in Amazonia [31]. In addition, a study conducted in the province of Granada found a tendency towards increased prevalence of melanoma at higher altitudes [32].

The present study also identified a significantly increasing AAPC over time for both sexes, particularly involving people under 50 years old. This rising incidence in younger people was unexpected. In fact, most cancers attributable to environmental causes tend to prevail in older age groups, and global epidemiological data [4,33] consistently associate a rising incidence of CMM with increasing age. To give an example, a population-based study from the UK (Office for National Statistics/Public Health England [34]) associated a higher incidence of CMM in both sexes with ages ≥ 64 years.

Based on these global and national trends, the rising incidence of CMM documented here in the younger residents of an Alpine area may suggest a combination of etiopathogenic mechanisms with both environmental and host-related risk factors at work [35]. A greater frequency of mini ozone holes has recently been reported across Europe, and they may affect large areas of the continent, including the Alps [16,17]. As for the potential host-related factors, a male predominance of CMM has consistently been documented, but an increasing body of evidence associates a significantly higher CMM incidence with younger females [31]. Beyond any behavioral differences between the two sexes (e.g., different clothing, different occupational activities, and different cultural habits) [36,37], the possibility of “cancer-prone” biological profiles cannot be excluded, and would find support in the different distribution of melanocytes and nevi in male and female bodies [38]. On these grounds, a major etiological role for cumulative sun exposure has been suggested for late-onset melanomas, while a melanoma-prone genetic profile could be a promoting factor in early onset disease [39].

This study also documented significant differences in the primary sites of CMM arising in the Alpine population, compared with the rest of the Veneto region. In particular, the prevalence of head and neck primary sites among the former was almost twice that of the regional population as a whole, while CMM involving the trunk was significantly less common (5.3% versus 12.5% in the whole Veneto population). These differences are basically in line with the current “biological” subtyping of melanocytic malignancies, which includes CMMs involving the face among those typically associated with cumulative solar damage (CSD) (Table 1) [40,41,42].

In our sample, cases of tumor regression and TILs were both more common among CMM patients living in the Alps. These findings potentially contrast with experimental data supporting anticancer immune suppression due to UV-B and UV-A spectra [14].

## 5. Limitations

This study has some limitations to consider. For one, no details were available about the patients’ lifestyles and occupations, and their cultural propensity to take preventive measures to protect their skin, as well as their molecular profiles, which could provide more insights into the observed phenomenon.

## 6. Conclusions

In conclusion, the present study documented an increase in the incidence rate of CMM and the associated AAPC in the Veneto region of Northeastern Italy over a period of nearly 30 years. This trend was particularly evident when the study focused on the population of the Alpine province of Belluno, where younger females showed the greatest rise in the incidence of CMM. A significantly higher prevalence of CMM involving the face, and a lower prevalence of cases involving the trunk, were also documented. These findings suggest the priority of establishing effective primary prevention measures in both sexes, particularly in the young Alpine population, where environmental genotoxic agents (solar radiation) may act in combination with genetic susceptibility.

At the same time, this study has highlighted the importance of constantly monitoring the trend of the incidence of cutaneous melanoma, and the need for a contextual comparison between areas with different geographical characteristics. In fact, there is great potential to apply geospatial approaches to various aspects of cancer prevention and control, in order to inform etiology and target interventions and the implementation of efficacious risk-reducing strategies [43]. Because health is shaped by factors beyond genetic susceptibility and clinical care, harnessing environmental exposure through geospatial approaches will allow for much better risk stratification of the population [44]. Some have called the community-based corollary “precision public health” [45]. Future studies will have to analyze the trend of the incidence of this disease, as well as of other cancer features, in other geographical contexts.

## Figures and Tables

**Table 1:** Average annual percent change ((AAPC); years 1990–2017) in the population of the Alpine province of Belluno versus the rest of the Veneto population, by sex and age group.

	CMM Patients’ Place of Residence	Age Group	Sex	
			Males	Females
AAPC				
	Alpine province of Belluno	All ages	5.7 ** (95% CI: 4.2–7.1)	4.4 ** (95% CI: 3.2–5.5)
	Other Veneto provinces	All ages	3.5 ** (95% CI: 3.1–4.0)	2.8 ** (95%CI: 2.4–3.1)
	Alpine province of Belluno	<49	6.9 * (95% CI: 4.2–9.7)	7.7 * (95% CI: 5.8–9.5)
		≥50	5.1 * (95% CI: 3.6–6.6)	2.3 * (95% CI: 1.0–3.6)
	Other Veneto provinces	<49	2.4 * (95% CI: 1.6–3.2)	2.7 * (95% CI: 2.1–3.3)
		≥50	3.9 * (95% CI: 3.4–4.4)	2.8 * (95% CI: 2.4–3.3)

Statistically significant. **: <0.001 *: <0.05.

**Table 2:** Different CMM histological variables distribution in the regional population as a whole, in the provinces other than Belluno, and in the Alpine province of Belluno. M: melanoma; TILs: tumor-infiltrating lymphocytes (Brisk is not considered).

	Veneto Region as a Whole	Veneto Region Excluding Belluno	Alpine Area (Belluno)	*p*-Value *
Age Mean (Std)	60.7 (16.0)	61.0 (15.8)	57.8 (18.2)	0.055
Sex N (%)				0.841
Male	726 (53.1)	672 (53.0)	54 (54.6)	
Female	642 (46.9)	597 (47.0)	45 (45.45)	
Primary site N (%)				**0.028**
Lower back	516 (39.7)	478 (39.6)	38 (40.4)	
Lower limb	292 (22.4)	273 (22.6)	19 (20.2)	
Upper limb	179 (13.8)	166 (13.75)	13 (13.8)	
Trunk	163 (12.5)	158 (13.1)	5 (5.3)	
Face	151 (11.6)	132 (10.9)	19 (20.2)	
M. histology Subtype N (%)				**0.044**
Superficial spreading	948 (69.3)	878 (69.2)	70 (70.7)	
Nodular	206 (15.0)	188 (14.8)	18 (18.2)	
Lentigo maligna	32 (2.3)	26 (2.05)	6 (6.1)	
Acral-lentiginous	23 (1.7)	23 (1.8)	0	
Desmoplastic	7 (0.5)	7 (0.55)	0	
Spitzoid	30 (2.2)	29 (2.3)	1 (1.0)	
Malignant (NOS)	122 (8.9)	118 (9.3)	4 (4.0)	
Growth pattern N (%)				0.350
Vertical	804 (74.9)	743 (75.3)	61 (70.1)	
Radial	270 (25.1)	244 (24.7)	26 (29.9)	
Breslow thickness N (%)				0.250
<0.75 mm	654 (51.05)	604 (50.8)	50 (53.8)	
0.76–1.50 mm	278 (21.7)	264 (22.2)	14 (15.05)	
1.51–3.99 mm	202 (15.8)	188 (15.8)	14 (15.05)	
≥4 mm	147 (11.5)	132 (11.1)	15 (16.1)	
Ulceration N (%)				1.000
Absent	1023 (80.3)	949 (80.3)	74 (80.4)	
Present	251 (19.7)	233 (19.7)	18 (19.6)	
Tumor regression N (%)				**<0.000**
Absent	630 (60.5)	601 (63.1)	29 (32.95)	
Present	411 (39.5)	352 (36.9)	59 (67.05)	
TILs N (%)				**0.007**
Present	862 (73.2)	791 (72.2)	71 (86.6)	
Absent	315 (26.8)	304 (27.8)	11 (13.4)	
Stage (TNM) N (%)				0.567
I	854 (67.1)	792 (67.1)	62 (66.7)	
II	215 (16.9)	202 (17.1)	13 (13.1)	
III	141 (11.1)	127 (10.8)	14 (15.05)	
IV	63 (4.95)	59 (5.0)	4 (4.3)	

* Pearson’s chi-squared tests or Fisher test and Student’s *t*-tests were used, respectively, to assess differences in clinicopathological variable distribution and mean ages between Alpine and Veneto region, excluding Belluno, populations.

## Data Availability

The data supporting the findings of this study are held by the Veneto Epidemiological Registry and were used under license for the present work, but they are not publicly available. These data are nonetheless available from Manuel Zorzi on reasonable request and subject to permission being obtained from the Veneto Epidemiological Registry (Veneto Regional Authority).
